# A case report of full recovery from severe cerebral edema secondary to acetaminophen-induced hepatotoxicity in a 13 year old girl

**DOI:** 10.1186/s12887-018-1233-5

**Published:** 2018-07-30

**Authors:** Emily B. Austin, Hailey Hobbs, Brian A. Crouse, Anna-Theresa Lobos

**Affiliations:** 1 Emergency Medicine, University of Toronto, Ontario Poison Centre, Toronto, Canada; 2 Department of Adult Critical Care Medicine, Western University, London, Canada; 3 Department of Pediatrics, University of Ottawa, Ottawa, Canada; 4 Division of Critical Care, Department of Pediatrics, University of Ottawa, Ottawa, Canada; 5 Department of Emergency Medicine, St. Michael’s Hospital, 30 Bond Street, Toronto, ON M5B 1W8 Canada

**Keywords:** Pediatric acute-liver failure, Cerebral edema, Acetaminophen-induced hepatotoxicity

## Abstract

**Background:**

Acetaminophen is a common cause of acute liver failure in pediatrics. Cerebral edema is a significant complication of acute hepatic failure and is associated with increased mortality.

**Case presentation:**

We present a case of a 13 -year old girl with severe cerebral edema secondary to acetaminophen toxicity and hepatic failure. Her poor neurological status precluded her from liver transplantation and withdrawal of life sustaining treatment was recommended. However, with supportive care, she remarkably made a full recovery.

**Conclusions:**

This case highlights the difficulties surrounding prognostication in pediatric patients with cerebral edema from acute liver failure secondary to acetaminophen toxicity.

## Background

Acetaminophen is the most common identifiable etiology of acute liver failure in the pediatric population in North America [1, 2]. Cerebral edema is a well- known end-organ complication of acute hepatic failure, and is associated with increased mortality [1].

We present a case of a 13 -year old girl with severe cerebral edema secondary to acetaminophen toxicity and hepatic failure. A poor neurological exam and significant cerebral edema precluded her from liver transplantation and withdrawal of life sustaining treatment was recommended. However, with supportive care she remarkably made a full recovery. This case highlights the challenges surrounding prognostication in patients with cerebral edema from acute liver failure secondary to acetaminophen toxicity.

## Case presentation

A 13-year-old Inuit girl (60 kg) presented to a remote nursing station in Arctic Canada. She had admitted to suicidal ideation and overdose, but was unable to disclose the time of ingestion or exposure. Family suspected that she had ingested clonidine and methylphenidate, thought to be the only medications accessible at home. She was otherwise healthy and had no prescribed medications. She had no known drug allergies and did not use alcohol, or any illicit substances.

On arrival to the nursing station, she was lethargic but rousable and followed commands. Her vital signs were stable, and she had a Glasgow Coma Scale (GCS) of 10. There was no evidence of meningismus or focal neurological deficits, and both cardiopulmonary and abdominal examinations were unremarkable.

Due to resource limitations, initial investigations were limited to point of care testing which included venous blood gas, electrolytes, glucose, creatinine, complete blood count and INR (international normalized ratio) (see Table 1). Results were notable for an elevated creatinine (2.32 mg/dL), elevated INR (5.0) and low serum bicarbonate (17 mmol/L). Alcohol, acetaminophen, salicylate level and chemistries including transaminase levels and lactate were not available.

**Table 1:** Laboratory results

	Presentation (Day 0, time 0)	24 h post-presentation	Arrival at pediatric tertiary care centre (38 h post presentation)	Arrival at pediatric quaternary care centre (46 h post-presentation)	Day 4 post-presentation	Day 10, 2 days prior to Transfer to Peds Ward	Normal Values
Serum APAP (umol/L)	NA	NA	249	112	NA	NA	
AST (IU/L)	NA	NA	5136	4728	677	84	8–45
ALT (IU/L)	NA	NA	9275	8688	3688	747	10–40
Bilirubin (umol/L)	NA	NA	28	13	16	9	0–17
NH4 (umol/L)	NA	NA	153	137	NA	NA	< 30
Cr (mg/dL)	2.32	2.08	1.03	0.58	0.67	0.49	0.63–1.10
INR	5	5.4	5.0	4.62	2.21	1.35	0.86–1.24
HCO3 (mmol/L)	17	19	22	21	31	29	20–25
pH (Venous)	7.37	7.34	7.36	7.43	7.42	7.43	7.35–7.41

*NA* not available

Two hours after her arrival, the nurses requested a transfer to the closest acute care hospital which was 2600 km away. While awaiting the transport team, she deteriorated clinically, becoming hypotensive (blood pressure 85/50) and neurologically unresponsive. Repeat neurologic assessment at 24 h after arrival had significantly worsened revealing nonreactive, dilated (6 mm) pupils and decerebrate posturing to pain. Available point-of-care laboratory testing was repeated (Table 1) revealing an INR was 5.4. An advanced care transport team (physician and respiratory therapist) arrived 28 h after presentation to the nursing station. Upon their arrival, they established an advanced airway and given the concern for increased intracranial pressure (ICP) based on the clinical picture, mannitol (500 mg/kg), phenytoin and ceftriaxone were administered. She received empiric treatment with vitamin K 10 mg IV (intravenous) for the elevated INR.

In discussions with the regional poison centre, the elevated INR and creatinine following overdose were suspicious for unrecognized acetaminophen toxicity. Aproximately 28 h after the patient’s initial presentation, N-acetylcysteine (NAC) was started empirically at a 140 mg/kg IV loading dose and continued at 70 mg/kg IV every 4 h.

**Fig. 1:**
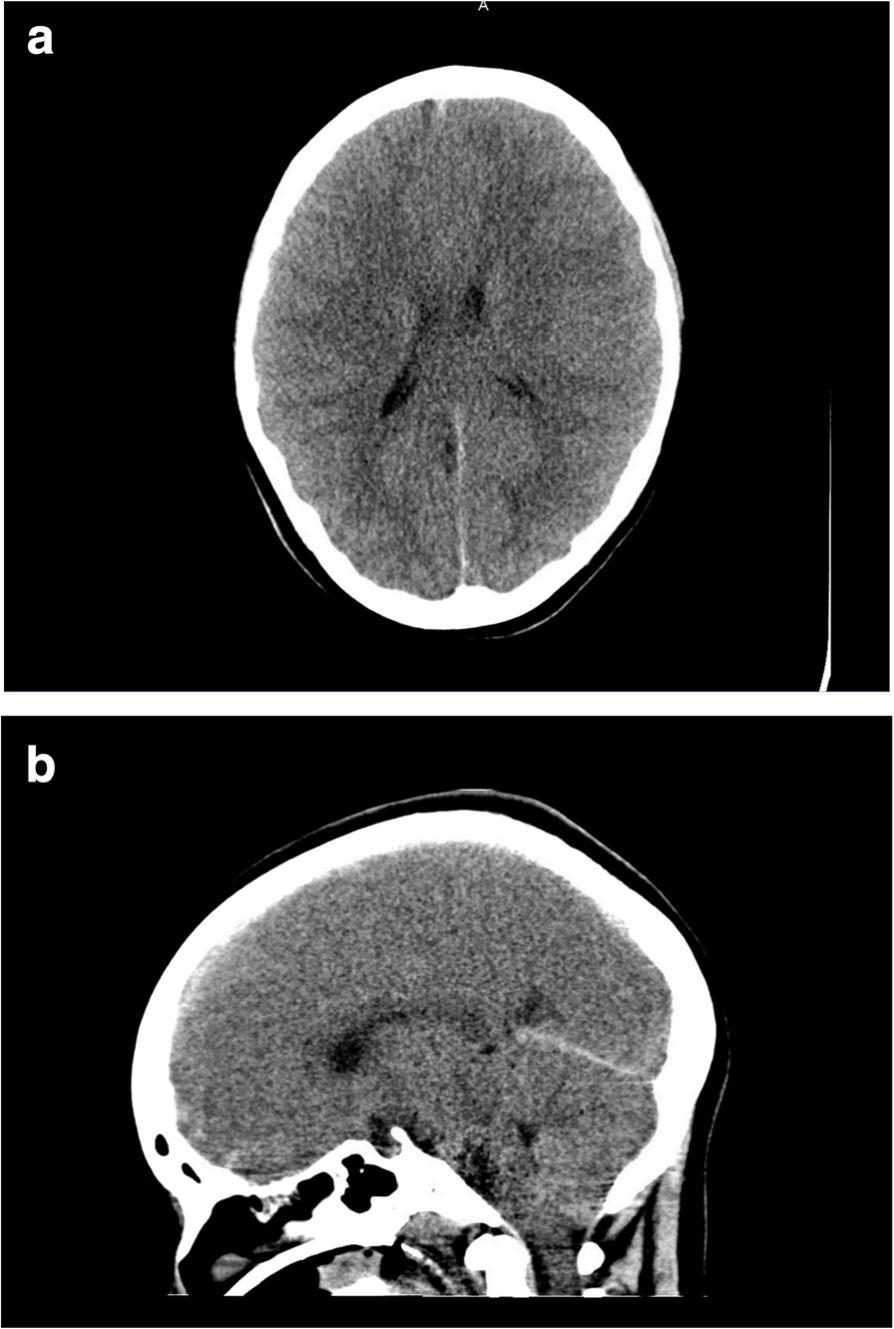
**a** Slice of CT brain showing cerebral edema. **b** Slice of CT brain showing suspected uncal herniation

The patient arrived at the pediatric tertiary care hospital 38 h after her initial presentation. Her neurologic examination revealed a GCS of 3 with pupils fixed and dilated 5 mm bilaterally, as well as intermittent posturing. Cough and gag reflexes were present. Laboratory investigations (Table 1) corroborated the suspected diagnosis of acetaminophen-induced acute liver failure. A CT (computed tomography) brain scan showed diffuse edema with effacement of sulci and basal cisterns, and uncal herniation (Fig. 1). Given these findings, she was transferred to a pediatric quaternary care center for evaluation for liver transplantation where she was assessed by neurology, neurosurgery and the liver transplant team. At 60 h post-initial presentation, her neurologic exam demonstrated fixed, dilated and non-reactive pupils, with flaccid tone in the upper extremities and increased tone in lower extremities, but without spasticity or clonus. She had a negative vestibulo-ocular reflex (no eye movement with “doll’s eyes” maneuver), absent gag and cough reflexes, but intact corneal reflexes. An EEG (electroencephalogram) showed continuous generalized slowing consistent with severe metabolic encephalopathy. Additional investigations to rule out alternative etiologies for acute liver failure included viral serologies, autoimmune and metabolic markers, and liver ultrasound, none of which revealed an alternative diagnosis. Given her poor neurologic prognosis, she was deemed ineligible for liver transplant and the withdrawal of life sustaining therapies was recommended.

The family agreed and requested that other family members be present prior to removal of life-sustaining therapy. Given the family’s request to wait a few days until others could arrive, she remained on full ventilatory support and continued to receive intensive care treatments, including NAC 70 mg/kg IV every 4 h until day 5, mannitol for neuroprotection (500 mg/kg IV), vitamin K (10 mg IV) and fresh frozen plasma (10 ml/kg) for coagulopathy. On day 4 her transaminases stabilized, and synthetic liver function studies improved. On Day 6, she spontaneously opened her eyes and lifted her fingers. She made continued neurologic improvements and 8 days post admission she was purposefully moving all extremities. With her remarkable clinical improvement, the care plan was changed from withdrawal of life sustaining treatments to full medical treatment. She was successfully extubated on day 11 following initial presentation and later continued recovery on the pediatric inpatient ward. She was discharged home 34 days following her initial presentation with a near complete neurologic recovery, save for a right third and sixth cranial nerve palsy.

## Discussion

Acetaminophen is the most common drug taken in overdose in pediatric and adult populations. Classically, clinical acetaminophen poisoning is described in 4 stages [3]. In the first 12–24 h after ingestion, patients present with non-specific symptoms such as nausea, vomiting and abdominal pain. After about 24 h, hepatic injury becomes apparent with elevated liver transaminase concentrations. In the third stage, patients can progress to fulminant hepatic failure, defined by the presence of encephalopathy and/or a coagulopathy (INR > 2.0). Patients will receive a liver transplant, recover, or die. Cerebral edema leading to cerebral herniation and ischemia is a common cause of death [4].

Our patient presented with acetaminophen–induced acute liver failure and evidence of cerebral edema. In addition to assessment for liver transplantation, management should focus on early initiation of NAC and supportive care, including ensuring adequate organ perfusion and treating increased ICP [5]. Unless there is an invasive procedure planned, coagulation factors are not given routinely to correct the INR [5].

While many healthcare providers consider acute liver failure-associated coagulopathy a contraindication for invasive ICP monitoring due to the risk of intracranial bleeding, a recent pediatric retrospective study of patients with severe hepatic encephalopathy associated with acute liver failure reported the use of invasive ICP monitoring and suggested that ICP monitoring provided additional information regarding central nervous system injury before liver transplant and allowed for directed treatment of ICP using osmolar agents [6]. Further studies are needed to explore the role and outcomes of invasive ICP monitoring in pediatric patients with severe hepatic encephalopathy associated with acute liver failure.

The decision to offer liver transplantation in an acetaminophen-poisoned patient is complicated in all cases, but especially in pediatrics. In adult patients with acetaminophen induced acute liver failure, the King’s College Criteria is a validated tool with acceptable specificity [7, 8]. Other prognostic tools include the MELD score, and the Acute Liver Failure Index [8, 9]. However, none of these tools are validated for use in pediatrics. Furthermore some authors suggest that pediatric patients may have improved ability of spontaneous liver regeneration. Ideally it would be beneficial to identify patients unlikely to recover without a liver transplant early in their presentation so that they would not develop devastating neurologic compromise. Almost exclusively, it is recommended that patients with cerebral edema *not* be considered for transplant [10].

Aside from the King’s College Criteria, there is a paucity of evidence to guide prognostication in patients with significant cerebral edema or herniation due to liver failure who are not transplant candidates. Alper et al. found that cerebral edema resulting from fulminant hepatic failure in a pediatric population was associated with a 70% mortality rate, with none of the patients demonstrating favourable neurologic outcome [11]. It is unclear if advances in the management of critically ill patients would have changed these outcomes today. Furthermore, the authors suggest, “termination of care is a reasonable option”. Kamat et al. reported a small retrospective review of intubated pediatric acute liver failure patients with grade III and grade IV encephalopathy [6]. In this study, 14% died prior to transplantation (2/14) while 28% (4/14) patients had spontaneous recovery without liver transplant [6]. A case report of a 4-day old neonate with acetaminophen-induced fulminant hepatic failure and presumed cerebral edema describes recovery without transplantation [12]. There are also reported cases of good functional outcome following cerebral herniation for prolonged periods in adults [13].

Without accurate prediction tools in pediatrics, physicians must make decisions based on prior experience. A retrospective study of an adult traumatic brain injury population found that increased mortality may result from decisions to withdraw life sustaining therapies due to the physicians’ perceived impression of poor prognoses [14].

## Conclusions

This case report highlights a situation where withdrawal of life sustaining therapies was recommended by two pediatric hospitals but not completed at the initial time of consideration as the patient’s family requested more time to allow other family members to be present. While waiting for family to arrive, she improved significantly and eventually made a complete recovery. Further studies in this area would be important to guide clinicians and potentially prevent withdrawal of life sustaining therapies in patients who may recover with usual intensive care.

## Abbreviations

CT: Computed tomography
EEG: Electroencephalogram
GCS: Glasgow Coma Scale
ICP: Intracranial pressure
INR: International normalized ratio
IV: Intravenous
NAC: N-acetyl cysteine
